# The accuracy of cystoscopy in predicting muscle invasion in newly diagnosed bladder cancer patients

**DOI:** 10.1007/s00345-023-04428-6

**Published:** 2023-05-17

**Authors:** Christine G. J. I. van Straten, Max H. Bruins, Siebren Dijkstra, Erik B. Cornel, Michael D. H. Kortleve, Thijn F. de Vocht, Lambertus A. L. M. Kiemeney, Antoine G. van der Heijden

**Affiliations:** 1 Department for Health Evidence, Radboud University Medical Center, PO Box 9101, 6500 HB Nijmegen, The Netherlands; 2 Department of Urology, Zuyderland Medisch Centrum, Heerlen and Sittard, The Netherlands; 3 Department of Urology, Canisius Wilhelmina Ziekenhuis, Nijmegen, The Netherlands; 4 Department of Urology, Ziekenhuisgroep Twente, Hengelo, The Netherlands; 5 Department of Urology, Ziekenhuis Gelderse Vallei, Ede, The Netherlands; 6 Department of Urology, Ziekenhuis Bernhoven, Uden, The Netherlands; 7 Department of Urology, Radboud University Medical Center, PO Box 9101, 6500 HB Nijmegen, The Netherlands

**Keywords:** Accuracy, Bladder cancer, Cystoscopy, Muscle invasion, Staging

## Abstract

**Purpose:**

The prognosis of muscle-invasive bladder cancer (MIBC) has not improved for three decades. Transurethral resection of the bladder tumor (TURBT) is the standard procedure for local tumor staging. TURBT has several limitations, including the spread of tumor cells. Therefore, an alternative is needed in patients with suspected MIBC. Recent studies have shown that mpMRI is very accurate in staging bladder tumors. Because the diagnostic efficacy of urethrocystoscopy (UCS) has been reported as good as the efficacy of mpMRI to predict muscle invasion we performed this prospective multicenter study in which we compare UCS with pathology.

**Methods:**

From July 2020 until March 2022, 321 patients with suspected primary BC in seven participating Dutch hospitals were included in this study. A flexible UCS was performed by urologists, physician assistants, or residents. Predictions of muscle invasion using a 5-point Likert scale alongside the histopathology data were recorded. The sensitivity, specificity, predictive values, and 95% confidence intervals were determined using a standard contingency table.

**Results:**

Of the 321 included patients, 232 (72.3%) received a histopathological diagnosis of non-muscle-invasive bladder cancer (NMIBC) and 71 (22.1%) were histopathologically diagnosed as MIBC. In 2 patients (0.6%), classification was not possible (Tx). Cystoscopy predicted muscle invasion with a sensitivity of 71.8% (95% CI 59.9–81.9), and a specificity of 89.9% (95% CI 85.4–93.3). This corresponds to a positive predictive value (PPV) of 67.1% and a negative predictive value (NPV) of 91.7%.

**Conclusion:**

Our study shows a moderate accuracy of cystoscopy to predict muscle invasion. This result does not support the use of cystoscopy only instead of TURBT for local staging.

**Supplementary Information:**

The online version contains supplementary material available at 10.1007/s00345-023-04428-6.

## Introduction

Muscle-invasive bladder cancer (MIBC) accounts for approximately 25% of all primary bladder cancer (BC) cases [1–3]. The European Association of Urology (EAU) guidelines recommend treating MIBC either with neoadjuvant chemotherapy followed by a lymph node dissection, radical cystectomy, and a urinary diversion or with trimodality bladder-sparing therapy in selected patients [4]. Despite the substantial improvements in disease management, the clinical outcome of the disease remains suboptimal, with an approximate 50% 5-year recurrence-free survival rate [4–7]. This outcome suggests the presence of unobserved micro-metastases before initiating radical treatment.

Transurethral resection of the bladder tumor (TURBT) is the standard procedure for local tumor staging. This is remarkable because such invasive techniques are not used for local staging in any other type of tumor. Indeed, small-scale studies have shown that during TURBT tumor cells are released [8–11]. These cells might induce the formation of micro-metastasis and thereby lead to a worse prognosis [12–14]. Furthermore, TURBT has a rather poor accuracy for identifying muscle invasion due to either insufficient tissue removal or inadequate tissue handling and assessment. Consequently, TURBT fails to recognize muscle invasion in approximately 30% of the cases [15, 16]. Finally, TURBT delays definitive radical treatment in patients with MIBC [17].

These limitations of TURBT support the importance of developing an alternative local staging strategy. One method that may be considered is the visual assessment of muscle invasion through cystoscopy because During et. al. reported cystoscopy to have a very high sensitivity of 92% and specificity of 88% to assess muscle invasion [18]. These impressive results would imply that an experienced urologist does not need a TURBT or another imaging method such as a multi-parametric MRI, combined with an outpatient biopsy for pathological confirmation, to diagnose MIBC. In this study, we aimed to replicate the high accuracy of cystoscopy to predict muscle invasion in newly diagnosed BC patients.

## Methods

### Subjects and study design

This study investigates the prediction accuracy of muscle invasion through flexible white light cystoscopy using histopathology as the reference standard. We included patients with suspected primary BC from seven collaborating Dutch hospitals between July 2020 and February 2022 (one hospital) and between May 2021 and February 2022 (the other six hospitals). Information on patient and tumor characteristics was prospectively collected from the medical files. According to the Dutch Law on Human Experimentation, this is a purely observational study (without any intervention or change in routine practice). This means that the study is exempt from approval by an ethics committee and that informed consent is not needed.

Urologists, physician assistants, and residents indicated whether they expected the concerning tumor to be muscle-invasive or not through the use of a subjective 5-point Likert scale: 1 = strongly agree the lesion is NMIBC, 2 = agree that the lesion is NMIBC, 3 = equivocal, 4 = agree that the lesion is MIBC and 5 = strongly agree that the lesion is MIBC [19]. Subsequently, the cystoscopic predictions were compared with the histopathology results from TURBT and re-TURBT to determine the accuracy of the predictions. Detrusor muscle had to be available in the (re)TURBT specimen. The TNM classification was used for staging, while the degree of differentiation was determined according to the 2004/2016 World Health Organization (WHO) classification [20–22]. In cases of re-resection, the highest T-stage was taken as the final result.

Tumors classified as PUNLMP (papillary urothelial neoplasm of low malignant potential) were regarded as low-grade Ta. In situations of benign/inflammatory lesions, a Likert score of 1, 2, or 3 was considered to be correct.

### Analyses

All analyses in this study were performed using R-Studio 2022.02. We considered a Likert score of 1–3 to be correct in situations of NMIBC. After all, hypothetically, in clinical practice, doubtful cases (Likert score 3) would primarily be managed as non-invasive in order to avoid unnecessary immediate radical treatment in false-positive cases. A subsequent analysis was performed in which patients with a score of 3 were added to the MIBC-predicted group to observe how the diagnostic accuracy would be affected if physicians would assume doubtful cases to be MIBC to avoid under-treatment (supplement 1).

The sensitivity, specificity, positive (PPV), and negative predictive value (NPV) were established using a standard contingency table that compares the ratios of true/false positives and true/false negatives. Confidence intervals for sensitivity and specificity were generated using the Clopper Pearson interval, while the confidence intervals for the predictive values were determined using the standard logit confidence intervals given by Mercaldo et al. [23, 24]. In two sub-analyses, we assessed the diagnostic parameters for urologists, residents, and physician assistants and the seven different hospitals.

## Results

Between July 2020 and February 2022, a total of 324 patients suspected of having primary BC were identified. Of these 324 patients, three cases were excluded. Two due to death before TURBT, and one due to missing histopathology data. Table 1 presents the baseline characteristics of the remaining 321 patients. 305 (95.0%) patients had malignant disease, while 16 (5.0%) were classified as having no malignancy as they were diagnosed with cystitis. 71 (22.1%) tumors were classified as MIBC and 2 (0.6%) were unknown (Tx). These two cases with unknown stages were excluded from the subsequent analysis. 64.2% of the UCSs were performed by urologists, 19.0% by residents, and 16.8% by physician assistants.

**Table 1:** Baseline characteristics of the study population

Variable	*N* (%)
Invasiveness	
NMIBC	232 (72.3)
MIBC	71 (22.1)
Unknown stage (Tx)	2 (0.6)
No malignancy	16 (5.0)
Tumor morphology	
Urothelial Bladder Cancer	291 (90.7)
Other morphology	14 (4.3)
Benign	16 (5.0)
Medical profession	
Urologist	206 (64.2)
Physician assistant	54 (16.8)
Residents	61 (19.0)
Hospital	
Institute 1	72 (22.4)
Institute 2	40 (12.5)
Institute 3	49 (15.3)
Institute 4	63 (19.6)
Institute 5	46 (14.3)
Institute 6	31 (9.7)
Institute 7	20 (6.2)

*NMIBC* non-muscle-invasive bladder cancer, *MIBC* muscle-invasive bladder cancer

The correlation between cystoscopy findings and pathological assessment is depicted in Table 2. Of all NMIBC cases, 73.7% were (correctly) scored with a Likert scale of 1 or 2. However, for T1 high-grade tumors, this percentage was only 47.8%. Of all MIBC cases, 71.8% were correctly scored with a Likert scale of 4 or 5.

**Table 2:** Correlation of cystoscopic findings and histologically confirmed diagnosis

Final histopathology	Likert scale 1–2 (%)	Likert scale 3 (%)	Likert scale 4–5 (%)
NMIBC	171 (73.7)	40 (17.2)	21 (9.1)
* Ta/LG*	100 (88.5)	11 (9.7)	2 (1.8)
* Ta/HG*	30 (73.2)	11 (26.8)	0
* T1/LG*	1 (100)	0	0
* T1/HG*	32 (47.8)	17 (25.4)	18 (26.9)
* Tis*	8 (80.0)	1 (10.0)	1 (10.0)
MIBC (T2–T4)	7 (9.9)	13 (18.3)	51 (71.8)
No malignancy	9 (56.3)	3 (18.8)	4 (25.0)

Likert score 1–3 is considered to be correct if NMIBC or no malignancy

Likert score 4–5 is considered to be correct if MIBC

*NMIBC *non-muscle-invasive bladder cancer, *MIBC* muscle-invasive bladder cancer

Table 3 presents the diagnostic parameters for the overall and sub-analyses where the predictions classified as dubious were regarded as NMIBC-predicted. The sensitivity to detect MIBC was 71.8% (95% CI 59.9–81.9%). The specificity was 89.9% (95% CI 85.4–93.3%). The PPV was 67.1% (95% CI 57.8–75.3%) indicating that one in three patients would be overtreated if decisions were based on cystoscopy only. The NPV was 91.7% (95% CI 88.4–94.2%). Our data further suggest that urologists underdiagnose patients more often than physician assistants and residents (lower sensitivity), but their PPV is somewhat higher (Table 3).

**Table 3:** Diagnostic parameters of cystoscopy for local staging of BC stratified by type of health care provider and hospital

*Statistic*	Sensitivity (%, [95% CI])	Specificity (%, [95% CI])	PPV (%, [95% CI])	NPV (%, [95% CI])
Overall (*n* = 321)	71.8 [59.9–81.9]	89.9 [85.4–93.3]	67.1 [57.8–75.3]	91.7 [88.4–94.2]
Function				
Urologist (*n* = 206)	64.4 [48.8–78.1]	92.4 [87.1–96.0]	70.7 [57.4–81.3]	90.1 [86.0–93.1]
Physician Assistant (*n* = 54)	95.0 [75.1–99.9]	88.4 [74.9–96.1]	62.4 [62.4–89.7]	97.4 [84.9–99.6]
Resident (*n* = 61)	80.0 [51.9–95.7]	82.6 [68.6–92.2]	60.0 [43.2–74.7]	92.7 [82.0–97.2]
Hospital				
Institute 1 (*n* = 72)	68.8 [41.3–89.0]	89.3 [78.1–96.0]	64.7 [44.6–80.7]	90.9 [82.8–95.4]
Institute 2 (*n* = 40)	100.0 [66.4–100.0]	93.6 [78.6–99.2]	81.8 [54.1–94.5]	100.0
Institute 3 (*n* = 49)	75.0 [34.9–96.8]	90.7 [79.7–96.9]	54.6 [32.2–75.2]	96.1 [88.0–98.8]
Institute 4 (*n* = 63)	35.7 [12.8–64.9]	97.1 [85.1–99.9]	83.3 [39.0–97.5]	79.1 [71.8–84.9]
Institute 5 (*n* = 31)	78.6 [49.2–95.3]	87.5 [71.0–96.5]	73.3 [51.4–87.7]	90.3 [77.2–96.3]
Institute 6 (*n* = 46)	75.0 [19.4–99.4]	80.8 [60.7–93.5]	37.5 [18.5–61.3]	95.5 [79.2–99.1]
Institute 7 (*n* = 20)	100.0 [54.0–100.0]	84.6 [54.6–98.1]	75.0 [45.6–91.5]	100.0

Likert score 1–3 is considered to be correct if NMIBC or no malignancy

Likert score 4–5 is considered to be correct if MIBC

*PPV* positive predictive value, *NPV* negative predictive value

## Discussion and conclusion

Cystoscopy followed by TURBT is the standard local staging procedure in bladder cancer. However, TURBT appears to have a few shortcomings. First, the presence of muscle invasion is missed in around 30% of high-risk cases because of inadequate sampling or reviewing of muscle tissue [16]. Second, TURBT represents an extra ‘step’ in the treatment pathway that contributes to delay in receiving definitive therapy, contributing to the poor survival of MIBC [25]. Third, TURBT has poor to moderate accuracy in determining variant histologies of bladder cancer [15]. Finally, during TURBT, tumor cells may be shed into the peripheral blood [8–11].

This study evaluates the cystoscopic accuracy in determining muscle invasion in an attempt to evaluate whether TURBT can be omitted. The most important conclusion of this study is that the PPV is not high enough to justify immediate radical treatment. The NPV is of lesser importance since false-negative evaluations would lead to a TURBT, which is the standard staging procedure anyway.

We believe that our study accurately reflects the ability of Dutch physicians to predict muscle invasion in bladder cancer patients because of the large number of cystoscopic assessments originating from seven hospitals in the Netherlands. Nonetheless, we cannot ignore some limitations of our study. First, the number of patients in some hospitals was fairly small (minimum of 20) and we cannot exclude some form of selection of patients by the physicians. Second, the Likert score is subjective as it is not based on any formal prespecified feature. Rather, the treating physicians need to go with their own interpretation of the scale. The inclusion of a middle-ground option (Likert score 3) may have undermined the validity of our results since physicians will tend to select this option to prevent unnecessary harm and procedures. On the other hand, the addition of this option does reflect daily practice where a TURBT is performed in all cases. Second, a relatively small number of cystoscopies were performed by physician assistants (*n* = 54) and residents (*n* = 61), which reduces the robustness of the diagnostic parameter estimates.

A limited number of previous studies assessed the clinical prediction of muscle invasiveness using cystoscopy at the time of diagnosis (Table 4). The majority of these studies were performed over 10 years ago [26–29]. The most recent study by During et al. (2016) reported cystoscopy to be an excellent predictor of muscle invasiveness with a sensitivity of 92% and a specificity of 88% [18]. This study used a 6-year-long inclusion period for a modest number of patients (*n* = 167) which raises the question of whether some kind of selection took place. The authors also did not report how they handled doubtful or challenging cases, which might have resulted in optimistic conclusions.

**Table 4:** Overview of studies concerning the accuracy of cystoscopy as a local staging tool

Study	Population	Location	Analysis/study objective	Findings	Comments/bias
During et al. [18]	167 primary BC cases: - 122 NMIBC - 45 MIBC	UK	Predictions of muscle invasion (5-point Likert scale), stage, and grade were compared to final histology after TURBT	Diagnostic parameters for predicting muscle invasion: - Sensitivity: 88.9%; - Specificity: 91.0%; - PPV: 78.4%; - NPV 95.7%	No report was made concerning the doubtful cases, which might have resulted in conclusions that are too optimistic The 6-year-long inclusion period yielded a relatively small number of patients
Cina et al. [29]	68 primary and recurrent cases: *- *61 NMIBC *- *7 MIBC	USA	Predictions of WHO/ISUP diagnosis and muscle invasion were compared to final histology after formal biopsy or TURBT	Diagnostic parameters for predicting muscle invasion: - Sensitivity: 57%; - Specificity: 88%	The mixed cohort with primary and recurrent tumors may have introduced bias
Mitropoulos et al. [28]	146 primary and recurrent cases: *- *104 NMIBC *- *23 MIBC - 19 benign	Greece	Predictions on the nature of the lesion (malignant or not), grade, and stage were compared to final histology TURBT	Agreement with histology (specialists): - Stage: 66.1% - Grade: 55.1% Agreement with histology (trainees): - Stage: 64.5% - Grade: 54.3%	The mixed cohort may have introduced bias
Herr et al. [27]	144 recurrent cases: - 143 NMIBC - 1 MIBC	USA	Predicted stage and grade were compared to final histology after TURBT	Agreement with histology: - pTa tumors: 94%; - Low-grade tumors: 93%	The histology of the primary tumor was not blinded from the assessors, which may have introduced bias

The study by Mitropoulos et al. (2005) evaluated the accuracy of using cystoscopy to predict both bladder tumor stage and grade. They concluded cystoscopy to be an insufficient staging method, given the low agreement between the prediction and the histological stage (66.1%) [28]. The results of this study, however, may have been biased by the inclusion of both newly diagnosed BC cases and recurrent cases. Knowledge of the primary tumor characteristics may influence the urologist’s judgment of the invasiveness of the recurrent tumor. Our study focuses only on patients suspected of having primary bladder cancer to avoid such potential bias. The study by Cina et al. (2001) introduced the same potential bias and calculated a 57% sensitivity and an 88% specificity [29]. Satoh et al. (2002) performed a multivariable analysis to evaluate which visual tumor characteristics facilitate accurate predictions concerning muscle invasion but, unfortunately, did not report any diagnostic accuracy parameters [26]. Herr et al. (2002) correlated the cystoscopic impression of both stage and grade with the final histology after TURBT. This study presented an impressive 94% agreement between the cystoscopic prediction and the final histology in pTa tumors and a 93% agreement in low-grade tumors [27]. However, their cohort consisted of solely recurrent patients whose previous tumor histology was not blinded from the assessors. This most probably influenced their conclusion about muscle invasiveness since low-grade non-invasive tumors seldomly progress on either criterion.

Our findings indicate some variation in the skills of the urologists, physician assistants, and residents, although the confidence intervals around the diagnostic parameters are very wide. The higher sensitivity (but lower PPV) for physician assistants and residents, may be related to the fact that they regularly consult with or being supervised by the urologist. The study by Mitropoulos et al. (2005), on the other hand, did not observe a difference between urologists and trainee cystoscopists [28].

Our study suggests cystoscopy is most accurate in ruling out muscle invasion, with a specificity of 89.9% and an NPV of 91.7% (Table 3). Cystoscopy seems to be less satisfactory when it is used to demonstrate muscle invasion with an overall sensitivity of 71.8% and a PPV of 67.1%. This does not provide sufficient confidence to start radical treatment as this would be overtreatment of one in every three patients. Our supplementary analyses in which we considered doubtful cases as MIBC led to an overall sensitivity of 90.1%, a specificity of 72.5%, a PPV of 48.5%, and an NPV of 96.2%. This would lead to even more false positives and potentially more overtreatment. Another staging procedure is, therefore, still required to establish the final stage of BC tumors.

Recently, a meta-analysis exhibited multi-parametric MRI (mpMRI) to have a sensitivity of 92% and a specificity of 88% in distinguishing invasive tumors from non-invasive tumors [30]. Accordingly, mpMRI is hypothesized to be a more reliable and potentially safer technique to stage bladder cancer locally. This offers a promising alternative to replace the current local staging paradigm using TURBT. The recently completed UK-based BladderPath study compared TURBT with mpMRI but did not define progression-free survival as a primary endpoint and lacks the power to conclude which staging technique is safer [17]. There is a clear clinical need to investigate whether mpMRI is as good as, or even better in staging bladder tumors.

To conclude, our study has shown moderate accuracy regarding muscle invasion using cystoscopy. Our results, however, do not support any practice changes in the local staging of BC at this moment. The findings call for a randomized controlled trial that will examine whether replacing TURBT with outpatient histological tumor confirmation combined with imaging such as mpMRI leads to better diagnostic parameters and higher progression-free survival. Such a trial is about to start in the Netherlands (the BladParadigm trial).

## Supplementary Information

Below is the link to the electronic supplementary material.

Supplementary file1 (DOCX 23 kb)

## Data Availability

The anonimyzed data are available upon request.
