# Supplementary material for: The accuracy of cystoscopy in predicting muscle invasion in newly diagnosed bladder cancer patients
Source: World J Urol. 2023 May 17;41(7):1829–35. doi: 10.1007/s00345-023-04428-6 (PMC10352162; doi:10.1007/s00345-023-04428-6)
Supplement: Supplementary file 1 — Supplementary file1 (DOCX 23 kb) [file 345_2023_4428_MOESM1_ESM.docx]

**Supplement 1**

Diagnostic parameters of cystoscopy for local staging of BC stratified by type of health care provider and hospital where Likert scale 3 is regarded as MIBC-predicted

| Statistic | Sensitivity  (%, [95%CI]) | Specificity  (%, [95%CI]) | PPV  (%, [95%CI]) | NPV  (%, [95%CI]) |
| --- | --- | --- | --- | --- |
| Overall (n=321) | 90.1  [80.7-95.9] | 72.5  [66.5-77.9] | 48.5  [43.1-53.9] | 96.2  [92.7-98.1] |
| **Function** |  |  |  |  |
| Urologist  (n=206) | 88.9  [76.0-96.3] | 74.7  [67.2-81.3] | 50.0  [42.9-57.1] | 95.9  [91.1-98.2] |
| Physician Assistant  (n=54) | 100.0  [69.2-100.0] | 72.1  [56.3-84.7] | 45.5  [34.0-57.4] | 100.0 |
| Resident  (n=61) | 86.7  [59.5-98.3] | 55.6  [38.1-72.1] | 29.4  [17.5-43.8] | 44.8  [34.9- 55.2] |
| **Hospital** |  |  |  |  |
| Institute 1  (n=72) | 93.8  [69.8-99.8] | 64.3  [50.4-76.6] | 42.9  [34.1-52.1] | 97.3  [84.2-99.6] |
| Institute 2 (n=40) | 100.0  [66.4-100.0] | 77.4  [58.9-90.4] | 56.3  [40.1-71.2] | 100.0 |
| Institute 3 (n=49) | 100.0  [63.1-100.0] | 77.8  [64.4-88.0] | 40.0  [28.8-52.3] | 100.0 |
| Institute 4 (n=63) | 85.7  [57.2-98.2] | 82.9  [66.4-93.4] | 66.7  [48.4-81.0] | 93.6  [79.9-98.1] |
| Institute 5 (n=31) | 78.6  [49.2-95.3] | 65.6  [46.8-81.4] | 50.0  [36.6-63.5] | 87.5  [71.3-95.2] |
| Institute 6 (n=46) | 75.0  [19.4-99.4] | 69.2  [48.2-85.7] | 27.3  [14.3-45.7] | 94.7  [76.4-99.0] |
| Institute 7  (n=20) | 100.0  [54.1-100.0] | 69.2  [38.6-90.9] | 60.0  [39.9-77.2] | 100.0 |

Abbreviations: PPV = positive predictive value; NPV = negative predictive value

*Likert score 1-2 is considered to be correct if NMIBC or no malignancy*

*Likert score 3-5 is considered to be correct if MIBC*
